# Prepared, Not Perfect: A Teaching Intervention to Improve Resident Doctors’ Confidence in Chest Drain Management

**DOI:** 10.7759/cureus.89906

**Published:** 2025-08-12

**Authors:** Najeeb Aftab, Joe Fayad, Alex Wilkins

**Affiliations:** 1 General Surgery, Mid Yorkshire Hospitals NHS Trust, Wakefield, GBR; 2 General Surgery, Hull University Teaching Hospitals NHS Trust, Hull, GBR

**Keywords:** chest tube, emergent general surgery, general thoracic surgery, junior doctor, patient safety-based medical education, postgrad medical education

## Abstract

Background

Intercostal chest drains (ICDs) are essential in managing pleural pathology, yet many resident doctors begin surgical placements without adequate preparation to manage them safely. National audits and educational literature continue to highlight this gap in procedural confidence and training.

Methods

This prospective quality improvement project evaluated the impact of a structured, resident-led teaching session on chest drain management. Eighteen newly rotating resident doctors in general surgery completed pre- and post-session questionnaires assessing confidence across 10 domains, including indications, contraindications, anatomy, equipment, technique, and ward-based troubleshooting. The session was delivered via a single, slide-based teaching module designed for scalability and ease of repetition. Results were analyzed using the Wilcoxon signed-rank test.

Results

Statistically significant improvements were observed in every domain (p < 0.001). Median confidence scores (5-point Likert scale) increased across all domains, with median shifts ranging from +1 to +3 points. The largest gains were seen in identifying indications for ICD insertion (confidence rose from 52% to 95%; median shift +3; effect size r = 0.88) and anatomical knowledge (43% to 90%; median shift +2; r = 0.85). Even in domains with smaller proportional increases, such as equipment familiarity (37% to 40%; median shift +1; r = 0.45) and drain adjustment (6% to 52%; median shift +1; r = 0.52), the effect sizes indicated meaningful practical improvements in confidence. More modest gains were seen in procedural skills such as drain adjustment and equipment familiarity, suggesting a need for additional practical reinforcement. A spider plot visually demonstrated consistent post-intervention improvement.

Conclusions

A single, focused teaching intervention significantly improved resident doctors’ confidence in managing ICDs. Given its low-cost, low-resource design, this model is ideally suited for integration into surgical induction across NHS trusts. While theoretical gains were substantial, future iterations should incorporate simulation or supervised practice to strengthen procedural competence and retention.

## Introduction

Few experiences are more unsettling for a newly minted doctor than standing alone on a surgical ward at night, faced with a hissing, bubbling chest drain and no clear idea of what to do next. For most Foundation Year 1 (F1) doctors, this scenario is not hypothetical; it is real. Despite the risks involved, junior doctors across the NHS are routinely expected to manage chest drains without having received any formal training on the subject [1-3].

This educational gap is striking, especially considering how critical intercostal chest drains (ICDs) are in both emergency and elective care [1,4]. Whether managing a pneumothorax, draining infected pleural fluid, or dealing with trauma, ICDs are a staple of surgical practice [4,5]. Yet in contrast to airway management or laryngeal intubation, which are taught early and reinforced often, chest drain insertion and management are rarely addressed in medical school or induction [6]. Structured training when entering clinical work is the exception, not the rule.

National reviews, including the Getting It Right First Time (GIRFT) respiratory medicine report, have identified wide variation in pleural procedure training provision across NHS trusts, with some offering no structured induction teaching [6]. The General Medical Council’s Promoting Excellence standards specify that doctors must be appropriately prepared for their clinical duties [7], yet procedural audits have shown that undertraining in chest drain insertion remains a persistent safety risk [3]. Across the NHS, there is no nationally mandated requirement for junior doctors to complete Advanced Trauma Life Support (ATLS) or formal bedside procedural training, including ICD management, before commencing surgical or medical rotations. The delivery and content of induction programs are determined locally, leading to substantial variation, with many providing no structured teaching on ICD management [6].

The absence of early, accessible teaching leads to an unsafe mismatch between clinical expectations and actual preparedness [7]. At our own trust, Mid Yorkshire Teaching Hospitals NHS Trust, junior residents are expected to manage chest drains as part of their general surgery rotations, often during nights or weekends. Without prior hands-on experience or even a basic teaching session, this becomes more than a learning gap; it becomes a safety issue [7]. And yet, it is rarely formally acknowledged.

For many of these trainees, the only comprehensive guidance available is the ATLS course, which does cover ICD insertion [8]. However, with costs often exceeding £1,000, ATLS is financially out of reach for many juniors [8], especially those who are not pursuing a surgical career. It is therefore entirely possible, and unfortunately common, for a doctor to work in a surgical unit without ever having been properly trained in a procedure as common and as high-risk as chest drain management.

It is also critical to acknowledge that ICD insertion is not a simple or “junior-level” procedure. It involves understanding thoracic anatomy, careful identification of safe insertion zones, and attention to equipment setup, sterile technique, and patient monitoring. As Henry et al. note, misplacement or inadequate drainage can worsen outcomes in conditions like pneumothorax [9]. Psallidas et al. describe iatrogenic injuries to the intercostal artery as serious but preventable complications that arise from poor technique [10]. Puchalski et al. further stress the need for caution even in cases with bleeding risk or abnormal clotting [11]. More recently, the European Respiratory Society emphasized the nuanced decisions involved in timing, drainage rate, and patient selection, further illustrating that ICDs are not “straightforward” tools [12].

It is therefore essential that doctors are adequately prepared to manage chest drains safely, even without specialist expertise [7]. This includes recognizing when a chest drain is not functioning correctly, understanding the distinction between Seldinger and surgical drains, and knowing when escalation to senior support is warranted. These are the hallmarks of a safe practitioner: prepared, if not perfect.

The evidence for structured education early on is strong [2]. Simulation-based teaching has been shown to improve both confidence and competence in chest drain insertion [13]. However, retention of procedural knowledge also depends on reinforcement and repeat exposure. One-off sessions are helpful but not sufficient.

To address this, we designed a resident-led educational quality improvement project (QIP) to improve ICD knowledge among surgical juniors. The delivery method was intentionally simple: a structured, presentation-based teaching session, covering core knowledge including indications, anatomy, equipment setup, troubleshooting, and removal. The session was designed for repeatability; it requires no simulation lab or costly equipment and can be delivered by senior residents across any NHS trust. The presentation model, already implemented in our department, is highly adaptable and could serve as a sustainable solution to this national training gap.

Ultimately, just as we would not expect a junior doctor to intubate a patient without training, we should not expect them to manage a chest drain without preparation. This QIP represents a step toward bridging that gap affordably, practically, and repeatably.

Objective

The objective of this study is to evaluate the impact of a structured, resident-led teaching session on junior doctors’ confidence in chest drain management, with the broader aim of enhancing patient safety through improved procedural knowledge and competence.

## Materials and methods

Study design

This prospective QIP was conducted at Mid Yorkshire Teaching Hospitals NHS Trust between November and December 2024. The project aimed to address the observed gap in knowledge and confidence among junior doctors regarding ICD management. It was registered with the local Clinical Audit Department and followed NHS guidelines for audit-based quality improvement. The project’s focus was on evaluating a structured teaching session delivered to Foundation Year (F1 and F2) doctors within the General Surgery Department at Pinderfields Hospital.

Participants

A total of 18 junior doctors (F1 and F2) participated in the study. All were newly rotating into general surgery and had not previously received structured education on chest drains during either medical school or induction. At our institution, 18 junior doctors rotate through the General Surgery Department every four months, and all eligible doctors were approached and agreed to participate (100% recruitment rate). This number reflects the average cohort size for the rotation. All participants were graduates of UK medical schools, and none had prior formal training in ICD management, ATLS, or related procedural workshops before commencing the rotation.

Educational intervention

The teaching intervention consisted of a single-session, presentation-based module led by senior surgical residents and delivered during the induction period at the start of the participants’ general surgery rotation. Its design was guided by the British Thoracic Society Pleural Disease Guideline (2023) and ATLS (11th Edition) to ensure clinical accuracy and adherence to national standards [4,8]. The content addressed key aspects of ICD management, including clinical indications and contraindications, thoracic anatomy, pleural physiology, insertion techniques (both surgical and Seldinger), equipment handling, troubleshooting, and criteria for safe removal. To enhance engagement, the session included small simulation-based practices such as hands-on familiarization with chest drain components, mock setup exercises, and troubleshooting of common equipment issues, providing participants with a practical link between theoretical knowledge and ward-based application [14,15]. The session design incorporated elements of adult learning theory by emphasizing interactive discussion and problem-solving.

Validation of the module was achieved through review by consultant surgeons and the Surgical Education Department at Mid Yorkshire Teaching NHS Trust, ensuring both clinical accuracy and educational relevance. Iterative feedback from the pilot sessions further enhanced the clarity and practical applicability of the content.

The module was piloted in two separate sessions of nine participants each, allowing for interactive discussion and real-time adjustments to content delivery. Feedback from the first session informed refinements to sequencing, particularly in the equipment and troubleshooting sections.

Assessment and data collection

To assess the impact of the teaching, participants completed an identical questionnaire immediately before and after the session. The questionnaire comprised 10 items using a five-point Likert scale ranging from “Strongly Disagree” to “Strongly Agree.” Questions evaluated perceived confidence and understanding across domains such as anatomy, physiology, equipment handling, procedural knowledge, and ward-based drain management. Data collection was conducted using Google Forms (Google LLC, Mountain View, CA, USA), with all responses anonymized.

Data analysis

Descriptive statistics were used to summarize pre- and post-intervention confidence scores across 10 assessed domains. Inferential statistics were applied using the Wilcoxon signed-rank test, appropriate for paired ordinal data, to evaluate the significance of observed changes. p-values <0.05 were considered statistically significant. All data tabulation and visualizations, including the spider plot, were performed using Microsoft Excel (Version 2506; Microsoft Corporation, Redmond, WA, USA). ChatGPT (OpenAI, San Francisco, CA, USA) was employed as an assistive tool for statistical summarization and figure preparation, with all outputs independently verified by the authors.

Ethical considerations

The project was conducted under the framework of local clinical audit and quality improvement governance. As such, formal ethical approval was not required. Anonymity and data confidentiality were maintained throughout, and no identifiable data were collected.

## Results

All 18 participating resident doctors (F1-F2 level) completed both the pre- and post-intervention questionnaires, yielding a 100% response rate. Analysis revealed substantial improvements in self-reported confidence across all 10 domains evaluated. These domains covered knowledge of indications, anatomy, physiology, equipment, insertion techniques, and ward-based management of ICDs.

Question 1: Confidence in identifying the indications for chest drain insertion

Before the session, 40% of respondents disagreed, and 37% remained neutral. Only 12% agreed and 11% strongly disagreed, indicating a widespread lack of confidence. Following the teaching, 75% agreed and 20% strongly agreed with the statement, while only 5% remained neutral. No participants disagreed post-session. This shift demonstrates a significant improvement in recognizing when chest drain insertion is appropriate.

Question 2: Confidence in identifying the contraindications for chest drain insertion

Pre-session responses showed 60% disagreement, 25% neutral, and 15% strong disagreement, revealing a major knowledge gap in understanding contraindications. Post-intervention, 55% agreed, 9% strongly agreed, and 23% remained neutral. Only 13% still disagreed. Although improvement was clear, a small proportion continued to express uncertainty, suggesting this area may benefit from reinforcement in future sessions.

Question 3: Knowledge of the anatomical landmarks for chest drain insertion

Prior to the session, only 15% of participants expressed agreement, with the majority split between neutral (40%), disagreement (28%), and strong disagreement (12%). After the session, confidence increased dramatically: 57% agreed and 33% strongly agreed. Just 7% were neutral, and only one respondent (3%) still disagreed. This result shows that anatomical orientation can be effectively addressed through structured teaching.

Question 4: Knowledge of the respiratory physiology

Participants reported mixed confidence levels pre-session: 28% agreed, 32% were neutral, and 40% either disagreed or strongly disagreed. Post-session, 72% agreed and 26% strongly agreed that they understood the relevant physiology. Only 4% remained neutral. This substantial shift demonstrates that foundational physiology, critical to interpreting drain function, can be effectively clarified with targeted instruction.

Question 5: Knowledge of the equipment necessary for insertion

Prior to the session, confidence in equipment familiarity was low: 28% strongly disagreed, 18% were neutral, and only 3% agreed. Post-session, responses were more evenly distributed, with 32% agreeing, 8% strongly agreeing, and 28% remaining neutral. However, 28% still disagreed, and 4% strongly disagreed. This domain showed the least improvement, highlighting a persistent knowledge gap in equipment setup that may require hands-on demonstration.

Question 6: Identification of the difference between Seldinger and surgical drains

In the pre-session questionnaire, 44% strongly disagreed, 28% disagreed, 12% were neutral, and only 16% agreed. Post-session, 64% now agreed with the statement, 8% strongly agreed, 8% were neutral, and only 26% still disagreed (including 4% strongly). This change illustrates that key procedural distinctions can be effectively taught even without simulation.

Question 7: Competence in insertion and removal

Confidence in this practical skill was lowest pre-intervention, with 44% strongly disagreeing, 36% disagreeing, 14% neutral, and just 6% agreeing. After the teaching session, 36% agreed and 32% remained neutral. Only 20% disagreed, and 12% strongly disagreed. Although notable improvement occurred, the persistence of neutrality and disagreement suggests that practical skill confidence requires more than didactic input, likely needing supervised practice or simulation.

Question 8: Identification of indications for removal

Before the teaching session, only 32% agreed, 28% were neutral, and 40% either disagreed or strongly disagreed. Post-session, 76% agreed, 16% strongly agreed, and only 8% disagreed. This represents a strong gain in knowledge regarding when a chest drain should be safely removed, a key step in ward-based patient care.

Question 9: Managing chest drains on the ward

Pre-session responses were heavily skewed towards low confidence: 36% strongly disagreed, 40% disagreed, and only 12% agreed or were neutral. Post-intervention, confidence improved substantially: 72% now agreed, 8% were neutral, and 20% still disagreed. This shift reflects the positive impact of the teaching session on practical, day-to-day management confidence.

Question 10: Adjustment of chest drains on the ward

The final domain assessed a more advanced aspect of management. Pre-session, half of the respondents (50%) strongly disagreed with their ability to adjust chest drains, 28% disagreed, and only 6% agreed. Post-session, 48% agreed, 4% strongly agreed, 16% were neutral, and 32% still disagreed. Although improved, this area remains a challenge, underscoring the complexity of troubleshooting and the likely need for real-time, case-based teaching or simulation.

Statistical summary of educational intervention results

Table 1 summarizes the pre- and post-intervention confidence levels for each assessed domain in chest drain management. Confidence scores reflect the percentage of participants who selected “Agree” or “Strongly Agree.” Statistical significance was assessed using the Wilcoxon signed-rank test for paired ordinal data. All domains showed significant improvement.

**Table 1:** Pre- and post-intervention confidence levels in chest drain management (Wilcoxon signed-rank test results) This table summarizes the percentage of participants who selected “Agree” or “Strongly Agree” for each confidence domain before and after the teaching intervention. The Wilcoxon signed-rank test was used to evaluate the significance of paired ordinal data. All domains demonstrated statistically significant improvements, with p-values <0.05 indicating meaningful gains in self-reported confidence. ICD, intercostal chest drain

Domain (question)	Pre-training confidence (%)	Post-training confidence (%)	p-value
1. Indications for ICD	52	95	<0.001
2. Contraindications for ICD	40	88	<0.001
3. Anatomical landmarks	43	90	<0.001
4. Respiratory physiology	28	98	<0.001
5. Equipment familiarity	37	40	0.003
6. Differentiating Seldinger vs. surgical drains	16	72	<0.001
7. Competence in insertion and removal	6	36	<0.001
8. Indications for removal	48	92	<0.001
9. Managing chest drains on the ward	12	72	<0.001
10. Adjustment of chest drains	6	52	<0.001

**Figure 1:**
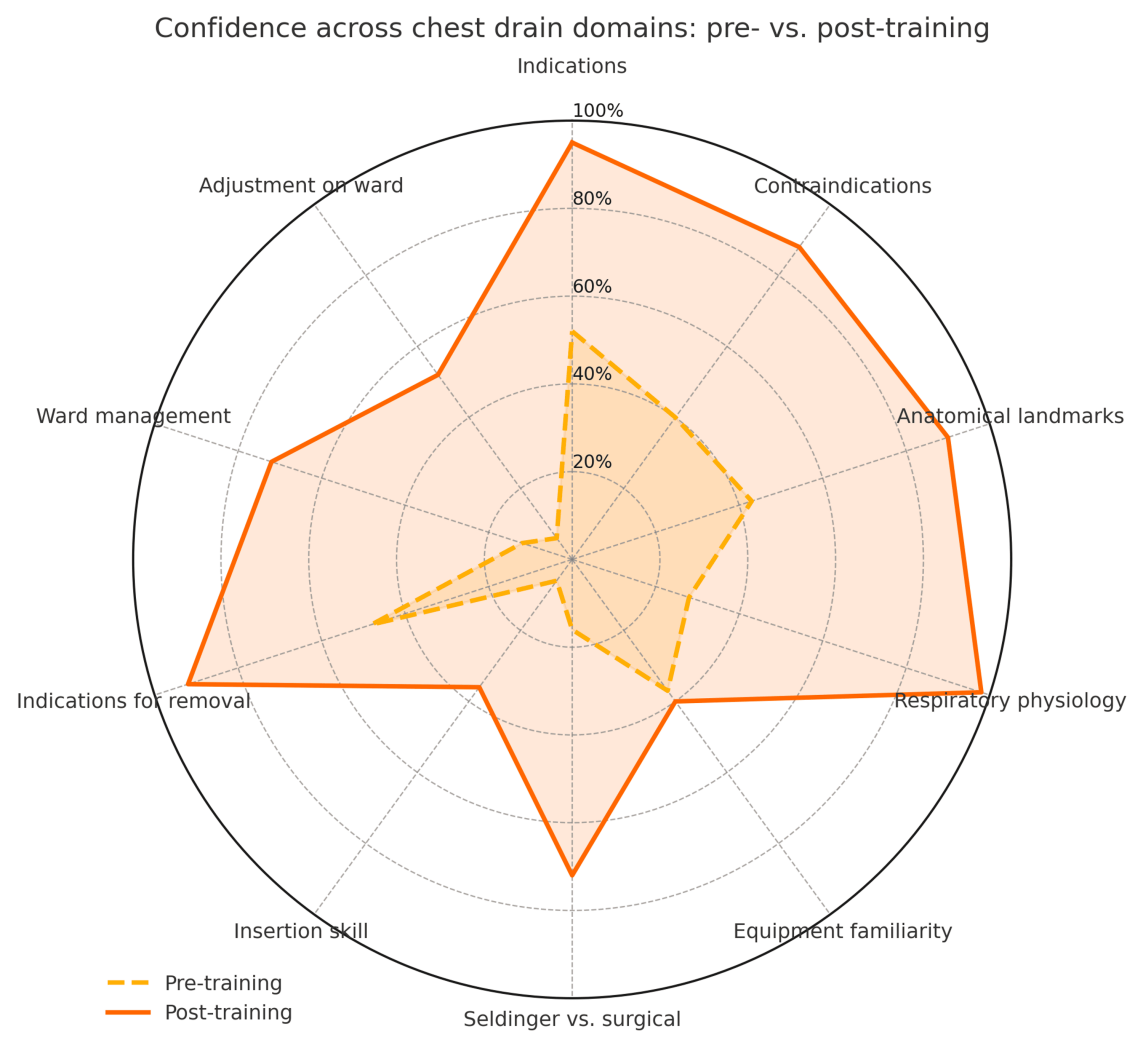
Spider plot of pre- and post-intervention confidence across 10 domains of chest drain management This figure illustrates the self-reported confidence levels of participants before and after the teaching intervention across 10 assessed domains. Each axis represents a key area of chest drain management, with values closer to the outer edge indicating higher confidence. The outward expansion of the post-training plot reflects consistent improvements across all domains, with the most pronounced gains seen in indications for insertion, anatomical knowledge, and ward-based management. The data are based on paired responses from 18 participants and analyzed using the Wilcoxon signed-rank test.

A radar (spider) plot was generated to visually compare confidence levels across all 10 assessed domains before and after the teaching intervention. The plot clearly illustrates a consistent outward expansion in the post-training scores, reflecting marked improvements in participant confidence across every domain. The most substantial gains were observed in anatomical understanding, indications for chest drain use, and ward-based management. While all areas improved, procedural domains such as equipment familiarity and drain adjustment remained relatively lower, reinforcing the need for supplementary practical training (Figure 1).

## Discussion

Our results tell a simple but powerful story: junior doctors can gain confidence and clarity in managing chest drains through a single, structured teaching session. Before the intervention, most residents were unsure, even apprehensive, about basic principles like identifying when a drain is needed or how to spot safe anatomical landmarks. After the session, we saw dramatic improvements across nearly every domain, particularly in indications for drain insertion (confidence jumped from 52% to 95%) and knowledge of anatomy (from 43% to 90%) (Table 1).

This is not surprising. Prior research consistently shows that junior doctors feel underprepared for pleural procedures [1,2]. Researchers found that new doctors often avoid these tasks altogether, citing a lack of formal teaching [1]. Our baseline data echoed that, with many participants expressing uncertainty about even the basic concepts. However, the shift post-teaching underscores a crucial point: the knowledge gap is real, but it is also highly addressable.

Not everything improved equally. Skills related to equipment familiarity and procedural insertion showed more modest gains, just 3% and 30%, respectively, even though the presentation covered these in detail. While the improvement in equipment familiarity was statistically significant, the absolute change was small. This may reflect the limitations of self-assessment in detecting subtle changes in procedural confidence, the modest sample size, and the likelihood that practical, hands-on experience is required to substantially shift confidence in this domain. These findings suggest that some skills cannot be taught with slides alone. Hands-on exposure or simulation may be essential for converting passive understanding into procedural confidence, as previous studies on simulation-based training have emphasized [13].

The spider plot (Figure 1) makes this pattern strikingly clear. Domains like anatomical understanding, indications, and ward management spiked outward dramatically, but areas like equipment setup and drain adjustment remained closer to the center [4]. This visual reinforces the idea that theoretical education is a great starting point, but it is not the whole answer.

What makes this project worth highlighting is its practicality. No simulation labs. No expensive models. Just a focused, well-structured teaching session led by residents. It is scalable, repeatable, and ready to plug into induction programs across the NHS. That is what makes this intervention not just effective but sustainable. The GIRFT report calls for exactly this kind of local, practical education to fill national training gaps [6]. More importantly, it aligns with the General Medical Council’s Promoting Excellence standards, which mandate that training providers ensure doctors are appropriately prepared for the clinical responsibilities they are expected to undertake [7].

Looking ahead, the next step should involve integrating Objective Structured Clinical Examinations (OSCE) or similar assessment frameworks to objectively measure skill acquisition and retention [14,15]. Additionally, the “hidden curriculum” plays an important role in how trainees acquire procedural confidence. Informal ward-based learning, often through observation or ad hoc supervision, can create variable and inconsistent experiences for residents [16]. Some trainees may gain confidence through frequent exposure to chest drain cases, while others may rotate through surgical placements without ever seeing the procedure performed. This variability underscores the need for structured, formal teaching sessions like ours to standardize learning, ensuring all trainees receive the same baseline knowledge and safety principles [16].

Poorly managed chest drains can cause life-threatening complications like intercostal artery injury or rapid pleural decompression [17]. By equipping doctors early with the basics, like what to do, what not to do, and when to ask for help. We are not creating experts; we are creating safer wards. Looking forward, we believe this teaching should be part of every surgical induction. However, we also recognize its limitations. Some skills require practice. Future iterations should pair teaching with simulation or supervised procedures. Checklists, refresher modules, and follow-up assessments would help embed this knowledge over time.

Limitations

This study has several limitations that should be considered when interpreting the findings. Firstly, the sample size was small and limited to a single NHS trust, which may affect the generalizability of the results. Secondly, the assessment relied on self-reported confidence scores rather than objective measures of procedural competence. While pre- and post-intervention questionnaires can demonstrate perceived improvement, they do not directly assess technical ability and are susceptible to response bias, including the Hawthorne effect. Objective measures such as structured multiple-choice question testing or supervised skills assessment were considered but could not be incorporated into this initial cycle due to time and resource constraints within the induction program. Thirdly, the evaluation was conducted immediately after the teaching session, providing no insight into long-term knowledge retention or the impact on clinical performance over time.

Additionally, the absence of a control group makes it difficult to rule out other contributing factors, such as concurrent ward-based exposure to chest drain management, which may have influenced confidence levels. Although minor simulation elements were included in the intervention, the session was primarily theoretical. Future studies should integrate structured simulation-based assessments, such as OSCE-style evaluations, to objectively measure skills and knowledge retention. Finally, as with any questionnaire-based study, the results are subject to response bias, which may have inflated self-assessment scores.

## Conclusions

A single, structured teaching session significantly improved junior doctors’ confidence in managing ICDs, particularly in clinical decision-making and anatomical understanding. While procedural skills showed more modest gains, this highlights the need for hands-on reinforcement alongside theoretical teaching.

Crucially, the intervention’s simplicity makes it both scalable and sustainable. Integrating such sessions into induction aligns with GMC requirements for safe practice and addresses a well-documented training gap. We do not expect mastery from new doctors, but we must ensure they are safe, supported, and prepared.

## Appendices

Appendix 1: Pre- and post-intervention questionnaire

The following questionnaire was administered immediately before and after the teaching intervention. Participants rated each statement on a 5-point Likert scale:

Strongly agree / Agree / Neither agree nor disagree / Disagree / Strongly disagree

1. I feel confident in identifying the indications for chest drain insertion.

2. I feel confident in identifying the contraindications for chest drain insertion.

3. I am able to accurately identify the anatomical landmarks for chest drain insertion.

4. I am aware of the physiological properties of the pleura and the mechanisms involved in ventilation.

5. I feel confident in identifying and preparing the equipment required for insertion of a chest drain.

6. I am able to identify the difference between a Seldinger drain and a “surgical drain.”

7. I feel competent in inserting and removing chest drains safely.

8. I am able to identify the indications for chest drain removal.

9. I feel confident in managing a chest drain on the ward that is leaking or not swinging.

10. I feel confident in identifying and adjusting a malpositioned chest drain on the ward.

The same items were used for both the pre-course and post-course assessments.

## References

[1] Wong CA, Lee O, Kennedy Y, Kenealy H, Hood C, Sivakumaran P, Lee YC (2009). The training, experience, and confidence of junior doctors in performing pleural procedures. N Z Med J.

[2] Aiyappan V, Munawar A, Thien F (2013). Junior doctor training in pleural procedures: a quality survey. Intern Med J.

[3] Elsayed H, Roberts R, Emadi M, Whittle I, Shackcloth M (2010). Chest drain insertion is not a harmless procedure - are we doing it safely?. Interact Cardiovasc Thorac Surg.

[4] Roberts ME, Rahman NM, Maskell NA (2023). BTS guideline for pleural disease. Thorax.

[5] Koegelenberg CF, Diacon AH (2013). Image-guided pleural biopsy. Curr Opin Pulm Med.

[6] Allen M (2021). Respiratory Medicine: GIRFT Programme National Specialty Report. https://gettingitrightfirsttime.co.uk/wp-content/uploads/2021/11/Respiratory-Medicine-Oct21L.pdf.

[7] Promoting excellence. GMC.

[8] American College of Surgeons Advanced Trauma Life Support. Advanced Trauma Life Support (ATLS) Student Course Manual. 11th ed. Chicago: ACS.

[9] Henry M, Arnold T, Harvey J (2003). BTS guidelines for the management of spontaneous pneumothorax. Thorax.

[10] Psallidas I, Helm EJ, Maskell NA (2015). Iatrogenic injury to the intercostal artery: aetiology, diagnosis and therapeutic intervention. Thorax.

[11] Puchalski JT, Argento AC, Murphy TE, Araujo KL, Pisani MA (2013). The safety of thoracentesis in patients with uncorrected bleeding risk. Ann Am Thorac Soc.

[12] Bedawi EO, Ricciardi S, Hassan M (2023). ERS/ESTS statement on the management of pleural infection in adults. Eur Respir J.

[13] Hutton IA, Kenealy H, Wong C (2008). Using simulation models to teach junior doctors how to insert chest tubes: a brief and effective teaching module. Intern Med J.

[14] Khan KZ, Ramachandran S, Gaunt K, Pushkar P (2013). The Objective Structured Clinical Examination (OSCE): AMEE Guide No. 81. Part I: an historical and theoretical perspective. Med Teach.

[15] Alharbi A, Nurfianti A, Mullen RF, McClure JD, Miller WH (2024). The effectiveness of simulation-based learning (SBL) on students' knowledge and skills in nursing programs: a systematic review. BMC Med Educ.

[16] Lempp H, Seale C (2004). The hidden curriculum in undergraduate medical education: qualitative study of medical students’ perceptions of teaching. BMJ.

[17] (2020). Deterioration due to rapid offload of pleural effusion fluid from chest drains. Dec.

